# Park pricing in theory and practice and implications for ecosystem and human health

**DOI:** 10.1016/j.eehl.2025.100151

**Published:** 2025-04-28

**Authors:** Krishnal Thirumarpan, Elizabeth J.Z. Robinson

**Affiliations:** a Department of Agricultural Economics, Faculty of Agriculture, Eastern University, Sri Lanka; b London School of Economics and Political Science, United Kingdom

**Keywords:** National parks, Park pricing, Health and well-being

## Abstract

Though a rich literature addresses the theory of park pricing, less attention has been paid to the practical realities. In this narrative review article, we ask why the setting of national park entry fees varies in practice, and we link this back to the underlying theory, the empirical academic literature, and practical realities. Park entry pricing strategies tend to differ considerably in higher and lower-income countries, reflecting practical realities of how to fund a national park system. Parks in higher-income countries are often free at the point of entry, consistent with the efficient pricing of global public goods. In contrast, differential pricing for local and foreign tourists is common in lower-income countries, an example of price discrimination that increases overall park revenues. We highlight a number of areas for further research. First, the concept of fairness and equitable access is an important practical consideration, linked to who benefits from visiting parks versus who pays, but much more attention needs to be paid to this in the literature. Second, while there is increasing recognition of the importance of green spaces for health and well-being, the literature largely ignores how health considerations might influence park entry fees, suggesting that more research is needed at the nexus of pricing, health and well-being, and equitable access. Finally, many lower-income countries that have a high dependence on foreign visitor fees to fund their national park systems are vulnerable to global shocks, suggesting research is needed into how to increase long-term sustainability of funding sources.

## Highlights

• Modalities for park entry fees vary across higher and lower-income countries.
• Charging entry fees reduces crowding and ecological pressure.
• Restricting access can increase health inequalities.
• Further research is needed at the nexus of entry fees, health, and equitable access.
• Long-run sustainability of funding is under-researched.

## Graphical abstract

**Figure:**
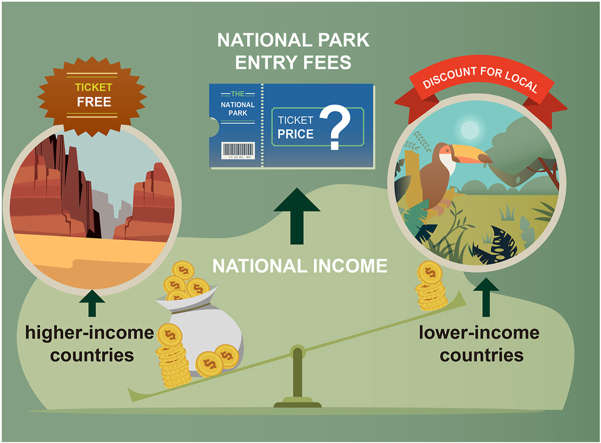

## 1 Introduction

Park pricing varies across countries. In some, all national parks are free to enter for all visitors; in others, all visitors pay the same park entry fee to visit; and in others, locals and foreigners are charged strikingly different amounts for the same experience. A considerable literature exists that addresses the theory of park pricing, in particular, whether or not countries should charge people to visit national parks; and whether differential pricing is appropriate [[1], [2], [3], [4]]. There is also a growing empirical literature that addresses the “optimal” park entry fee. Whilst some of the earlier literature focused on the fee that maximises revenues from charging tourists to enter the park, increasingly, analyses incorporate consideration of the broader impacts of an entrance fee on ecosystem services and environmental degradation, and other revenue generating opportunities for people living and working near the parks, each of which is likely to affect a more broadly defined “optimal” entrance fee.

Despite a rich and growing literature, there is much less attention paid to the extent to which the practicalities and realities faced by countries reflect and are reflected in the literature. Recognising this, the objective of this review article is to contribute to the literature by exploring how the theory behind whether park entrance fees should be charged and at what level compares with the practical realities and challenges faced by countries as they attempt to balance often competing demands placed on national parks, such as to provide, for example, government revenue; national and global public goods, including biodiversity conservation and health; and educational opportunities [5,6]. We highlight different pricing strategies that can be found in practice across a number of high- and low-income countries and several key areas where academic research effort might be focused in the future.

## 2 Methods

Methodologically, our paper is centred around a narrative review, which ensures we have space to provide interpretation and critique and deepen understanding [7]. A narrative approach is also sufficiently flexible to ensure that we consider the topic in question from a broad and exploratory perspective, synthesising across both academic literature and practical policy documentation, and allowing for an iterative approach to determining which documents are included in the review.

There are three distinct elements to our narrative review. The conceptual framing comes from the theoretical underpinnings of park pricing, which spans the extent to which a national park has attributes of a public or club good; why parks might or might not charge an entry fee; and the potential impacts and implications of the choice of park entry fee. We then consider both the early and more recent empirical literature that has tended to focus on an “optimal” entry price, generally in low and middle-income countries where charging an entry fee is more common. The third component of our review is to document practical examples of park pricing in a number of specific countries, and to explore the rationale behind pricing strategies in these countries. This focus on practical aspects of park pricing, and the linking back to theory and forward to areas for future research is, we feel, a particularly novel contribution.

For the conceptual framing and theoretical underpinnings, we started our literature search with the search terms “national parks” AND (“public goods” OR “club goods”) to scan the academic literature in Scopus and Google Scholar. We did not include any date restrictions, searching all entries up to the end of 2024. Given the large number of articles returned, in Scopus we therefore restricted the search to title, abstract, and/or keywords, which yielded 22 articles, and in Google Scholar we adjusted the search to terms “national parks” AND “public goods” AND “club goods”, but even this restriction yielded 499 articles from Google Scholar. We therefore searched purposively, targeting highly cited papers that could be considered seminal for this area of investigation and that focus on the underlying theory of externalities and public and club goods in the context of national parks, with articles selected to be included in this review that illustrate key specific aspects of the underlying theory.

In addressing the empirical literature, we used the following search terms in Scopus, with the number of articles returned in brackets: “national park” AND “pricing” (63), “national park” AND “entry fees” (16), “national park” AND “user fees” (14), “national park” AND “health benefits” (28), “national park” AND “well-being” (289), and “national park” AND “entrance fee/user fee” AND “health/well-being” (3). We supplemented this search by using the same search terms for Google Scholar. Given the large number of articles returned, we again focused primarily on highly cited articles and those that particularly related to the theory and practice of park pricing.

Finally, to document practical examples of how governments set entry fees, we first scanned the articles identified above. We then searched through the grey literature to identify key relevant policy documents. In addition, we identified a number of websites or newspaper articles that contribute to providing a richer understanding of the practical aspects of decisions over charging park entry fees that national park authorities must often take into account. Because almost 100 countries have national parks, we selected examples that illustrated well the points raised in the theoretical and empirical literature. It could be argued that to some extent this has resulted in a somewhat ad hoc selection, so the country examples we give should be seen more as case studies than broadly representative, the purpose of which is to motivate further exploratory and rigorous research that has clear policy relevance.

In total, our three searches resulted in over 500 publications relevant to this narrative review, including journal articles, working papers, books, technical papers, theses, and grey literature. Given the restrictions we placed on our search, we recognise that even this set of publications is a small subset of a much broader set; yet, equally, is too large a set to review in detail. We therefore reduced the number of articles to be included in our review article yet further. If the title and abstract of a peer-reviewed publication, or the text of a grey literature article, fitted the broad remit of focusing on park entry fees in the context of revenue generation, equity, and health, the full text was read, and the publication was saved. Out of the initial search, we refer to 70 publications explicitly in this review, which has allowed us to craft a compelling narrative and inform future research directions.

## 3 Conceptualising national parks as club or public goods

National parks have variously been cast as club goods, national public goods, and global public goods. There are a number of arguments that have been made in the literature as to why national parks might be best classified as public goods, which in the main are focused around the idea that they provide a “service which, if supplied to one person, can be made available to others at no extra cost” [8], and that they are non-rival and non-excludable in use, in which case, they cannot be valued directly [9,10]. Parks may also be considered as national public goods, in as much as they provide benefits such as improved physical and mental health, recreational facilities, water catchment enhancement, and natural hazard mitigation at a local rather than global level [11]. To the extent that a national park is conceptualized as a public good, once that park has been established, in theory, anyone should be able to use and enjoy it without cost, because the marginal cost of adding one more visitor would be zero [12]. Specifically, the assumption of non-rivalry in the context of a national park implies that the benefits gained by one individual will not negatively affect other visitors. Based on this rationale, the optimal pricing strategy from a social welfare perspective would be a zero-entry fee for all visitors.

In practice, conceptualising national parks as public goods is reasonable, up to a point. When few people visit a park, they might reasonably be assumed not to detract from others' own park experiences. However, the reality is that each individual tourist is likely to impose some small direct cost on the park in terms of administration and management, and some small externality cost in terms of crowding and degradation of the ecosystem and health benefits. This would imply that at the least a small “token” entry fee should be charged. At some point overcrowding will almost certainly reduce the enjoyment of all those visiting the park, whether due to degradation of the park ecosystems, noise, or simply the number of people in the park [13]. As such, many authors suggest that national parks are generally better considered as club goods [10,14,15], which are non-rival and excludable goods except for congestion [16].

There are some aspects of national parks that can be considered to be global public goods, such as biological diversity and genetic heritage, and climate change mitigation and carbon sequestration, because the global population benefits from the presence of the park [11,17,18] The concept of global public goods came into popularity in the second half of the 20th century [19]. A key issue for resources that are conceptualized as global public goods is how to fund the protection and management of these goods, particularly if they are located in one country, but citizens of all countries benefit [18,20]. Opinions towards these two views are mainly centered around such issues as use value versus non-use value and efficiency versus equity [1,3]. Governments, through decisions over whether and how to make the parks excludable, and whether and at what level to charge for entry, determine whether their national parks are treated more as public goods, free at the point of use for all; or “user pays” club goods where people must pay to enter and enjoy, and entry numbers may be limited. National parks are made excludable if there are entrance gates that visitors must go through where they are asked to pay an entrance fee. Some countries may prioritise the protection of natural resources and therefore explicitly choose to exclude people entirely from some areas, for example, to conserve historic and highly endangered resources for future generations to also enjoy. If the aim of conservation includes a consideration of future generations' enjoyment and health, then treating a national park as a club good rather than a pure public good may be appropriate [16].

## 4 Insights from the empirical park pricing literature

The question of how much, if at all, a government should charge people visiting its national parks is not a new one. The theoretical underpinnings were rigorously addressed in the 1990s and early 2000s. Some of the earlier empirical literature focused on the relatively narrow question of the optimal park entry fee that maximise revenues from those entry fees, but this literature has broadened considerably to encompass differential pricing, and broader landscape considerations.

Dikgang and Muchapondwa [4] suggest that there are four objectives linked to choices over whether to charge a park entry fee, and what level that fee should be: imputing value to visitation; ensuring parks are managed at economically efficient levels; promoting social equity that might include health considerations; and ensuring ecological limits are not breached by considering how entry pricing can be used to manage visitor numbers and reduce crowding [21,22]. However, there is considerable evidence in the literature that price elasticity of demand for national parks tends to be low, and as such considerable increases in entry fees would be needed to reduce visitor numbers substantially. Further, without price discrimination, which is increasingly widespread, sufficiently high entry fees, particularly in lower-income countries, are likely to price local visitors out of the market, making the parks only accessible to foreign tourists and the highest-income local households [23].

Gregersen [24] identifies three different pricing mechanisms: token charges, going rate charges, and cost-based charges. The author suggests that tokens, or small charges, have negligible impact on overall demand, and are too low to raise significant revenues, but have the additional benefit of establishing a pricing policy. An alternate explanation for token charges is that they can be used to impute value to an attraction [25]. Going-rate charges are described as reflecting the idea “that pricing of a given nature-based attraction should be equivalent to charges at comparable attractions after adjusting for differences in site quality, travel costs, visitors' incomes and other demand factors” [24].

Several studies find that, even where nature-based tourism sites get public funds for management, they still struggle with inadequate funds for maintenance [26]. In many such cases, park entry fees are often seen primarily as a practical way of raising much-needed funds to manage the infrastructure and upkeep of a park. A “user pays” perspective further recognizes that a considerable number of individuals never visit national parks, and it might be considered unfair to charge these people indirectly through general taxation. Manning et al. [27] suggest that in contrast to public education, recreation in natural sites is not mandatory for the public, and not all the residents of a country benefit from national parks. The implication of this is that all tourists who are using this service should be charged an entrance fee. A public good perspective suggests that the enjoyment and health benefits gained from a public good must be free for all, thereby improving the whole nation's welfare. This implies that the cost of conservation might be contributed to by the public of that nation through government taxation [3].

### 4.1 Optimal park entry fees

In addition to the choice over whether or not to charge an entry fee is the decision over how high that fee should be. Following Becker [28], numerous academic studies that address the optimal park entry fee have explored the feasibility of increasing park entry fees to maximise entry fee revenue for a particular national park or a specific country. Indeed, Dikgang and Muchapondwa [4] suggest that most park visits are underpriced, in as much as tourists have stated that they would be willing to pay more. They further add that if higher-income country tourists are willing to pay more to visit national parks, at the then prevailing prices, these tourists are being subsidised to visit lower-income country parks. Stevens et al. [29] suggest that for those national parks in the US that charge entrance fees, increasing the fees would have only a small impact on the number of visitors, which implies that there is considerable scope for increasing entry fees and total revenue, but this raises issues of just how high a fee would need to be to manage pressure on the ecosystem through entry pricing alone, and the ensuing implications for equity.

Various methodologies have been used to determine the revenue maximising entry fee, including travel cost methods and choice experiments. A study in Maasai Mara national park in Kenya, using the individual travel cost method found the optimal conservation fee to be US$ 86.90 per day, higher than the entrance fees at the time [30]. A study in Komodo national park, Indonesia, found that a fivefold increase in entrance fee would add substantial revenue to the government for the conservation of parks, but would not significantly affect the visitor numbers [31]. Dikgang and Muchapondwa [4] found that in South Africa, the revenue maximising entry fee was 115% greater than was being charged at the time, and that this higher fee would almost double the revenue from park entry fees, even allowing for a reduction in the number of people visiting the park. However, such a strategy might exclude lower-income households who in general have less access to green spaces and the associated health and well-being benefits, suggesting increasing health inequalities as a result.

These early contributions to the academic literature on park pricing made important contributions to the empirical literature but could be limited in cases where they focus on just one aspect of park revenue, that which accrues to the park through entry fees. A more holistic approach takes into account the impact of park entry fees on other revenue-generating activities, including accommodation occupancy, spend in restaurants, and additional spending, such as on souvenirs. Higher entry prices, by potentially reducing both the number of visitors and spend per visitor, could reduce the total spend of tourists in and around a park, or in the destination country more broadly. This could, for example, be detrimental to those local communities located near to a national park that may benefit from providing meals, services, and souvenirs for tourists passing through on the way to the park. Mukanjari et al. [32] found that for Kruger National Park in South Africa, consumer surplus per day for international tourists varies from US$346 to US$644, suggesting that there is considerable scope for parks to capture at least some of this surplus, through not just adjusting the entry fee but also pricing for the full suite of activities that visitors engage in.

As such, a national park authority that takes into account revenues from park entry fees, tourism spend in and around the park, health and well-being improvements that accrue to visitors, and ecosystem benefits that increase as the number of visitors falls, might set an entrance fee that was higher or lower than the fee that simply maximises revenues from entry fees. Location-specific research would be needed to determine whether increased revenues from higher entry fees and additional ecosystem benefits from fewer visitors outweighed fewer visitors' additional spend and fewer visitors gaining health and well-being benefits from visiting the park.

Yoon and Zou [33] caution that there is still insufficient understanding of how entrance fees affect visitor experiences and revisit intentions. Their research, focused on the US, suggests that higher entry fees could reduce visitor expenditure during a visit and reduce the likelihood that an individual revisits a park. In practice, determining an “optimal” entry fee is yet more complex. Local and international visitors, particularly those visiting parks in lower-income countries, are likely to have very different demand functions. Moreover, how and whether individual countries would and should account for local and international consumer surplus also complicates the definition and determination of a conceptual “optimal” park entry fee. That said, it is clear that studies that focus only on the impact that an entry fee has on total entry fee revenue are almost certainly over or underestimating the optimal fee, depending on the particular park circumstances.

Whilst many empirical papers address the “optimal” park entry fee, often finding it to be higher than the prevailing fee, fewer papers have addressed the very practical question of how to introduce or increase a park entry fee. One exception is Willis [2], who suggested that political barriers to implementing an admission fee for the Bosco di Capodimonte National Park in Naples, Italy, could be overcome by introducing a low initial entry fee, which is increased over time incrementally, in addition to differential pricing, with a lower price for locals and lower-income people. Another exception is Wilson and Tisdell [23], who explored attitudes towards the introduction of entry fees for a park in Australia when entry was currently free. Tourists were found to be more willing to pay if the revenues raised would be used to benefit the specific park and its visitors, and foreign tourists were more likely to support an entrance fee than Australian visitors than local Queensland visitors.

Another area where less attention has been paid is the extent to which the introduction of a park entry fee for one national park can affect visitor rates at other national parks. Exceptions include Chase et al. [34] and Melstrom and Vasarhelyi [35]. More recently, Shoji et al. [36] developed a Kuhn-Tucker model to explore how introducing an entry fee of 1000 JPY, approximately US$7, to just one of Japan's national parks affects the other 33 parks in the country. They find that remote parks are less affected, whereas easier-to-access parks with lower travel costs are more affected.

### 4.2 Differential pricing

There are clear rationales presented in the literature as to why differential entry pricing is reasonable and/or efficient. For example, Becker [28] compares four pricing strategies: free entrance, maximum revenue pricing, cost recovery pricing, and differential pricing, and concludes that a differential pricing system is the best option in terms of cost-effectiveness and reducing the dead-weight loss. Alpízar's theoretical model suggests that third-degree price discrimination based on a visitor's nationality can be justified by appealing to distributional fairness linked to assigning welfare weights linked to the consumer surplus of different visitor groups [37].

Where lower fees are paid by local visitors, the justification tends to be in part because the local population bears the opportunity cost of alternative uses of the parkland, and also, many residents already pay domestic taxes that may in part fund the establishment and maintenance of parks, when those costs are not covered by entry fees and other related charges [22,32,38]. In contrast, overseas visitors pay taxes in their home countries, so an entrance fee can be seen as a way of recovering from them the benefits they gain from visiting the national parks [3]. Overseas visitors to national parks in lower-income countries also typically have a higher willingness to pay as they are more likely to come from a higher-income country. The empirical evidence suggests that low- and middle-income country (LMIC) governments are more willing to focus on foreign rather than local tourists as an important source of revenue generation. More broadly, there is an argument that foreign tourists who gain the advantage of using another country's public resources should pay for the conservation of those resources [39]. Indeed, if LMIC governments do not differentially price, then residents of these countries could be seen to be subsidising visits from tourists coming from higher-income countries [24].

A number of the early studies on park pricing have confirmed different willingness to pay between local and international tourists. These include Shultz et al.'s study of repeat visits to two different Costa Rican National Parks [40], Maharana and Sharma's India study [41], and Asafu-Adjaye and Tapsuwan's willingness-to-pay for scuba diving in a marine national park in Thailand [42]. A study by Wilson and Tisdell [23] on introducing a minimal entry fee to Lamington National Park, Queensland, Australia, found that foreign tourists were willing to support the user fees more than locals. A contingent valuation study comparing the willingness to pay of tourists with that of, residents and administrative staff in Qianjiangyuan National Park System, China, found that tourists were willing to pay more than residents and administrative staff [43].

## 5 Pricing strategies in practice

In this section, we explore the practical realities of park pricing through select examples of countries' choices over how and at what level to set park entry fees. We note that ultimately, park entry price policies can be seen as political decisions, reflecting the choices of governments as to whether they are aiming to maximise government revenues from the national parks, maximise social welfare, address equity conditions through differential pricing, or simply cover the costs of maintaining the parks [2].

### 5.1 Entrance fees and practical implications for overcrowding and equity

Many higher-income countries do not charge people to visit national parks. For example, in New Zealand, legislation states that national parks cannot charge entry fees, and that the public has freedom to enter and access the parks without charge (National Parks Act 1980). Similarly, in the UK and France, national parks are free at the point of entry, funded through general taxation [44]. Nordic countries also tend not to charge national park entry fees [3]. In the US, at the time of writing, just 108 national parks (of a total of 417) charge an entrance fee.

By not charging people to enter their parks and not limiting numbers, countries are implicitly treating their parks as public goods, with equitable access to all who are able to visit and gain the health and well-being benefits from those visits. However, in some countries where there is currently no charge, whether to charge entrance fees for national parks remains contested. This appears particularly so when there are explicit concerns over overcrowding. In such cases, national parks have a number of broad alternatives that variously include rationing access to the park; or reducing numbers through other means that either make it more difficult or more costly to visit the park, each of which has equity implications.

One particular example is Iceland, which does not charge people to enter its national parks. Rather, conservation and management costs of natural attractions have been managed using tax revenue. Over time, higher numbers of tourists visiting the country's national parks have resulted in crowding that has imposed increasing costs on the government. This led some to suggest that entrance fees be charged so that visitors would bear at least some of the costs of conservation and management, thereby reducing the burden on governments to find sufficient funds [3,26]. Yet despite these concerns from some locals, there remained in Iceland quite broad local resistance to entry fees being introduced. Consequently, the country opted for a compromise of sorts, including charging parking fees at the most popular sites, and increasing concession fees [45]. This relatively recent innovation is in line with the early academic literature that suggested approaches to generating park revenue through alternatives to entry fees, including user fees for visitor services such as camping sites and boat and guide hire, and special taxes near parks such as for rooms or excise taxes for outdoor equipment [46]. Iceland's three national parks currently remain free to enter, and only one of the national parks charges to park a car, with the somewhat nominal cost set at 1000 ISK, just over US$7 (https://www.iceland.org/geography/national-park/; https://www.icelandtravel.is/attractions/thingvellir-national-park-2/).

Overcrowding in US national parks has recently been highlighted in the press and the academic literature, particularly in the summer season. For example, Hobbs et al. [47] estimated that if Zion National Park, where there have been particular concerns over visitor numbers, were to increase its fee per vehicle from the then prevailing US$35 to US$70 during this peak period, visitor numbers would fall by 18%.

There have been various more general considerations in the US over whether to increase entry costs to cover the costs of maintaining facilities. For example, in 2017 there were discussions to increase entry fees for some parks from US$25 to US$70 for a week-long pass [48]. The rationale was that the revenues were needed to renovate and restore the park infrastructure. Arguments against the proposed price increases tend to address the public good aspect of the parks, that there should be equitable access to parks, regardless of ability to pay, so fees should not be increased, and indeed some argue that all fees should be eliminated. American national parks that do charge an entry fee do offer occasional “free entrance days” to ensure that all people can visit the parks without this cost, and as such address, albeit to a limited extent, equitable access to the parks.

Entrance to Spanish national parks is free, though there have been on-going discussions as to whether a tourist or “ecotax” should be charged for tourists visiting protected areas. In the Spanish Cíes Islands, concerns over the impact of high tourist numbers on the environment were addressed through rationing. In 2017, the government limited the number of visitors to 1800 per day. In 2024, the government has decided to reduce that cap further to 450 visitors per day during the high season between mid-May and mid-September [49]. More broadly, higher-income countries that historically have not charged people to enter and enjoy their national parks are increasingly considering and implementing user fees [36].

China provides an interesting case study because, though the country's first nature reserve was established in 1956 [50], it is only recently in 2011 that the country conceptualised a national park system [51], and in 2015 issued *the Scheme for Establishing National Park System Pilot*, focused on protecting natural ecosystems and cultural heritage [52]. As has been the same in many other countries, the issue of whether to charge an entrance fee, and what the level of that fee should be, has been an important part of the planning conversations [52]. Experts have researched different national park systems around the world, including the United States model which has influenced the establishment of the Chinese National Park system [51]. Studies have been conducted in Chinese national parks to assess the willingness to pay for different parks [[52], [53], [54]].

Sri Lanka has always charged visitors to enter the national parks. As far back as 1997, researchers have observed that both local and international tourists in Sri Lanka were willing to pay considerably more to visit the country's national parks than they were being charged through entrance fees. Specifically, Silva and Kotagama [55] found that local tourists were willing to pay an entry fee of Rs. 69.50 to visit Udawalawe National Park, whilst at the time the actual fee was Rs. 18, and that this higher entry fee of Rs. 69.50 would increase revenues by over 200%, even allowing for fewer visitors as a result of the increase in fee. The authors suggest these findings reflect a considerable undervaluation of wildlife viewing in the country. However, it is important not to conflate low entry fees, or an absence of entry fees, with the “undervaluation” of national parks. The authors also determined that an increase in entry fee by over 200% would result in visitor numbers falling by around 47% and that this could also have ecological benefits. In Yala at that time there were already concerns over overcrowding, and that the social and environmental carrying capacities of tourists might already have been breached [56], especially as no restrictions were in place to limit visitor numbers. An increase in entry price would therefore be expected to bring both increased revenue and additional ecological benefits. However, a reduction in local visitor numbers due to increased entry fees would likely have had considerable implications for equitable access, and could be interpreted as Sri Lanka evolving its national parks from national public goods to club goods that only the better off could afford. Given that it is increasingly recognised that there are health benefits associated with visiting green spaces, increased access restrictions for lower-income households to national parks through increased fees might also increase health inequalities, another important trade-off to take into account.

### 5.2 One-price and differential entry fees

Where countries do charge entrance fees for national parks, these charges are often addressed in terms of practicalities or concepts of fairness. In those higher-income countries that do charge a park entry fee, there is typically no differential pricing. For example, in the US, in parks that do charge a fee, all non-concessionary visitors pay the same amount. The situation is generally quite different in lower-income countries, where differential pricing is much more common, and generally manifested as different entrance fees being charged to locals and foreigners [28]. Residents and citizens might be charged a nominal entry fee, whilst the fee for foreign tourists is much higher. Perhaps not surprisingly, in lower-income countries, foreigners tend to be more willing to pay more than locals, which enables park managers to increase total revenue through price discrimination compared to a one-price entrance fee, and by extracting rents from higher-income tourists [57].

A comprehensive survey by Van Zyl et al. [22] identified 51 countries that charge entry fees for both citizens and international tourists. They found that parks in Benin are the least affordable for citizens; and Tanzania's parks charge on average the highest fees for international tourists [22]. At the start of 2021, a non-East African tourist paid US$60 per day to enter Tanzania's world famous Serengeti National Park (vehicle charges are extra), an expatriate US$30, and a local around US$4.34 (10,000 Tanzania shillings) [58]. These fees were increased in July 2021 to US$71.80 for foreign tourists [59].

The issue of differential pricing has also been raised in some higher-income countries, particularly where the parks are free at the point of entry. In New Zealand, for example, there have been reports that some locals feel foreign tourists should pay entry fees to contribute to the upkeep of park facilities, whilst others continue to argue that all people should have “unfettered access to wilderness areas” [60].

### 5.3 Earmarking park entry fees

In some countries, park entry fees fully or partially stay within the specific park; in others, park entry fees are pooled, and in others, fee revenue goes directly to the central government. Earmarking revenues creates strong aligned incentives for parks to increase tourism numbers, but it is likely that, for example, there are some parks with few visitors but high ecological value that would not be able to be self-funded.

In France, though there is currently no charge to enter a national park, the introduction of entry fees has reportedly been considered as a way to provide a dedicated revenue stream to cover park maintenance costs. In Sri Lanka, revenues from park entry fees and park accommodation directly contribute to the government consolidated fund. Each year the government allocates a certain percentage of this consolidated fund to park management.

When Yellowstone National Park, the US's first national park, was created in 1872, there was an agreement that the park would be self-funded and not require any funding from Congress. However, as Fretwell and Podolsky [61] note, from the beginning the US army was responsible for law enforcement and management of the park, and by the early 20th century national park revenues went to central government funds. However, approaches to funding changed once again, and now in the US, all entry fees remain within the National Park Service, with 80% or more staying in the park where it was collected [62], suggesting a move towards earmarking entry fee revenue. Indeed, the Federal Lands Recreation Enhancement Act (FLREA) requires that park entry fees are used to enhance the visitor experience.

International organisations such as the World Bank and the Global Environment Facility (GEF) also provide funding to Sri Lanka based on the different proposals submitted by its Department of Wildlife Conservation. Out of the total income received from national parks, 50% is allocated to the wildlife preservation fund and the remaining 50% is paid to the respective provincial councils for infrastructure development in that particular area [63]. As such, entry fee revenues collected by a particular park are not earmarked for a specific park, rather funding is allocated based on the determined needs of each park.

## 6 Discussion and implications for future research

Pricing strategies for national park entry fees vary across countries, reflecting multiple perspectives on, for example, whether parks should charge entry fees at all, and if so how high those fees should be; and the extent to which a country can afford to protect its national parks through general taxation rather than earmarking user fees. This paper is motivated by theoretical discussions in the literature over whether national parks are best characterized as public goods or club goods, as well as empirical explorations of optimal park user fees. It addresses the extent to which the theoretical and empirical literature relates to and informs the practical realities of governments choosing whether and how much to charge people to visit their national parks.

To the extent that a national park can be considered a public good, there are both efficiency and equity arguments that access should be free at the point of entry. Yet the reality is that governments, particularly in lower-income countries, may not be able to prioritise funding national parks from general tax revenue when there are many other pressing demands, so charging entry fees and earmarking those fees become important practical policy considerations. Furthermore, visitors impose costs on parks and other visitors, so entry fees can be used to reduce visitor numbers and therefore visitor externalities, as an alternative to restrictions being placed on the number of entry permits allocated.

Our exploration of the literature and different countries' approaches to park pricing suggests that the rich and elegant literature on the theory of park pricing does not always match the realities of park pricing strategies that are complex, messy, nuanced, and country specific. The practical realities of charging entry fees for national parks make clear that parks cannot be classified as one particular type of good or service, but rather the pricing strategy itself very much determines whether a park is a de facto global public good, a national public good, or a club good, and the equity implications that accompany pricing choices. For example, in many higher income countries, where national parks are free for all to enter, as is the case for many of the parks in the US, the parks become de facto global public goods. In contrast, in lower-income countries with differential entry fees, a nominal fee for locals casts national parks as somewhat close to national public goods for the country's residents, but club goods for foreigners who pay a much higher fee to enter.

Despite the rich and expansive literature on the theory and practice of park pricing, there remains considerable scope for further studies that address important policy implications of funding of, and access to, countries' national parks; and that take into account the practical realities of how countries set user fees to access nature. We propose several promising areas for future research linked to equity, health, and the long-term sustainability of sources of revenue generation.

First, all choices over pricing access to national parks have equity implications. Pricing affects demand and therefore the extent of congestion and overcrowding [64], but also highlights important policy implications as national park services will need to balance equity considerations, such as ensuring broad access regardless of ability to pay, with practical concerns over ecosystem degradation and revenue generation. The concept of equity comes up a lot in the literature, both as an argument for national parks being free at the point of entry, and for national parks charging entry fees. Our detailed exploration of several individual country rationales for whether or not to charge an entry fee makes clear that some residents want their national parks to be open and free to all. This ensures that people can visit the parks irrespective of ability to pay, notwithstanding the costs of getting to the park, and can be interpreted as an equitable access argument. Yet we found evidence that some residents argue that those who benefit from visiting a national park should pay, and that it is unfair for those who do not want to visit a national park, do not see value in the park, or who are unable to visit, to still pay for the parks through general taxation.

Equity is a normative concept, linked to ideas of fairness and justice, and some literature focused on protected areas and conservation suggests that an equity framing might be particularly appropriate to guide further research into park pricing and user fees more broadly [65,66]. Particularly in lower-income countries, differential pricing enables parks to charge foreign tourists relatively high fees, whilst ensuring access for local tourists through nominal fees. This could be perceived as an equitable approach, as foreign visitors to national parks tend to have a higher willingness to pay, and they do not support these parks through their taxes. National park services and governments thinking of introducing or increasing park entry fees might therefore consider undertaking studies to determine local and national attitudes towards user fees for the national parks, using an equity and justice lens, in addition to the more common studies that attempt to determine the optimal park entry fee.

Given that the reality remains that many national parks are already overcrowded, some type of rationing of local tourists might be needed. How to ration entry, without pricing lower-income households out of the market, is increasingly important, given the better understanding of the links between nature and well-being. Future research might explore how a combination of limiting visitor numbers, differential pricing across space and time of year and visitor type, and linking this to encouraging people to visit less popular parks, might reduce the trade-offs inherent in managing competing demands for revenue, protecting nature and biodiversity, health, and equity.

Second, we suggest that there is still plenty of scope for further studies that explore the impact of park fees on the broader landscape around a national park that comprises economic development, livelihoods, and ecosystem services. Considerable attention has been paid in the literature to determining the “optimal” park entry fee that maximises total revenue from those fees, and the almost inevitable conclusions are reached that park entry fees are too low, and higher fees would bring in greater and much needed revenues. Though increasing park fees also has the benefit of reducing congestion, and reducing the pressure on the broader ecosystem, people living near to the park may lose out on important revenue generating opportunities, given that there are fewer people visiting the parks who also may have less income to spend once the higher entry fee has been paid.

Third, there is a separate literature that explores the health benefits of national parks and protected areas more broadly [[67], [68], [69], [70], [71]]. However, we have found that the park pricing literature is much less likely to consider the human health benefits of access to national parks, and how this might affect the empirical determination of an optimal user fee and governments' practical choices over entry fees. Future empirical research that explores and quantifies the health benefits of national parks could contribute to supporting policy makers that want to include benefits for human health from visiting national parks when considering park pricing strategies. For example, introducing a user fee, or increasing the user fee, can make parks less financially accessible for low-income households, which might be those that would get the greatest health benefits from being able to access nature. Indeed, a government concerned with equitable access to green spaces such as national parks should arguably also be factoring broader health benefits, given the increasing recognition of the importance of access to nature for health and well-being.

Finally, we feel that very little attention has been paid in the literature to the long-term sustainability of revenue sources that fund national park systems, especially for lower-income countries that tend to rely heavily on charging relatively high park entry fees for overseas visitors, rather than general taxation. Setting a high price for overseas visitors who tend to come from higher-income countries and therefore have a higher willingness and ability to pay brings in important revenue for lower-income countries. However, this could make national park services in these countries particularly vulnerable to local and global shocks. An important question, all but missing in the literature, is how revenue shortfalls can be made up if, for some reason, overseas tourists no longer visit, whether this is due to financial crises, natural disasters, or violent attacks [72,73]. Sri Lanka has twice experienced just such a situation in the past decade. First, after the Easter Sunday attack in April 2019, and second during COVID in 2020 and 2021. Each time tourist numbers fell dramatically. In Uganda in 1999, eight tourists were killed when visiting Bwindi Impenetrable Forest National Park, famous for its gorillas, and where non-resident tourists currently pay US$800 to go gorilla tracking. It took several years for tourism numbers and the associated revenue to recover, not just in Bwindi, but also in other national parks in the country [74]. Future research of importance to policy makers could explore to what extent lower-income countries were able to continue to manage and protect their parks during COVID, extreme weather that cuts off access to a park, conflict, or other shocks, and whether more diverse funding sources might be needed in the future to build resilience to such situations, that may well be more common in the future [75].

## 7 Conclusion

In this paper we sought to explore the extent to which governments' practical choices of whether to charge people to visit national parks, and if so how much, appear to be informed by the theory of park pricing, in particular focused on public and club goods, and empirical analyses that tend to focus on what an optimal park entry price might be, generally in the context of the potential to increase park entry fees from their current levels.

Our paper has explored the evolution of this theoretical and empirical literature, highlighting some of the key articles that have framed the various relevant debates. But more so, informed by the grey literature, and focusing on practical user fee choices made by national park authorities and governments, it has illustrated how governments must often make pragmatic decisions, including how to ensure equitable access that is compatible with conservation imperatives, long-term sustainable funding sources, and the increasing recognition of the importance of green spaces for human health and well-being. Researchers are well placed to provide the empirical evidence base and analysis for park authorities to make well-informed decisions that explicitly address both efficiency and equity.

## CRediT authorship contribution statement

**Krishnal Thirumarpan:** Writing – review & editing, Writing – original draft, Methodology, Funding acquisition. **Elizabeth J.Z. Robinson:** Writing – review & editing, Supervision, Methodology, Conceptualization.

## Declaration of competing interests

The authors declare that they have no known competing financial interests or personal relationships that could have appeared to influence the work reported in this paper.
